# Acne Detection Based on Reconstructed Hyperspectral Images

**DOI:** 10.3390/jimaging10080174

**Published:** 2024-07-23

**Authors:** Ali Mohammed Ridha, Nor Ashidi Mat Isa, Ayman Tawfik

**Affiliations:** 1 Department of Electrical and Computer Engineering, Ajman University, Ajman P.O. Box 346, United Arab Emirates; ali.mohd@student.usm.my (A.M.R.); a.tawfik@ajman.ac.ae (A.T.); 2 School of Electrical & Electronic Engineering, Engineering Campus, Universiti Sains Malaysia, Penang 14300, Malaysia

**Keywords:** acne detection, hyperspectral imaging, hyperspectral reconstruction, RetinaNet, machine learning

## Abstract

Acne Vulgaris is a common type of skin disease that affects more than 85% of teenagers and frequently continues even in adulthood. While it is not a dangerous skin disease, it can significantly impact the quality of life. Hyperspectral imaging (HSI), which captures a wide spectrum of light, has emerged as a tool for the detection and diagnosis of various skin conditions. However, due to the high cost of specialised HS cameras, it is limited in its use in clinical settings. In this research, a novel acne detection system that will utilise reconstructed hyperspectral (HS) images from RGB images is proposed. A dataset of reconstructed HS images is created using the best-performing HS reconstruction model from our previous research. A new acne detection algorithm that is based on reconstructed HS images and RetinaNet algorithm is introduced. The results indicate that the proposed algorithm surpasses other techniques based on RGB images. Additionally, reconstructed HS images offer a promising and cost-effective alternative to using expensive HSI equipment for detecting conditions like acne or other medical issues.

## 1. Introduction

Acne Vulgaris (common acne) is a type of skin disease that occurs when dead skin cells clog hair follicles [1]. It often affects skin with a high number of oil glands, usually the face, but it can spread to involve other parts of the body. Acne can be non-inflammatory (blackheads and whiteheads) or inflammatory (papules and pustules).

Although acne is not a dangerous skin disease and is sometimes thought of as unimportant, it can leave scarring, which has a major impact on people’s quality of life [2,3] and it can lead to significant levels of anxiety and depression. Acne affects more than 85% of teenagers [4] but frequently continues into adulthood [5,6], and it is consistently included in the top three most prevalent skin conditions in the general population [7,8,9]. Studies have shown that early identification and treatment of patients who have acne is vital and may improve their quality of life [10,11].

Hyperspectral imaging (HSI) is an imaging technique that captures a wide spectrum of light. In contrast to traditional RGB imaging, which uses primary colours (red, green, and blue) to represent each pixel in an image [12], HSI breaks down the light hitting each pixel into numerous spectral bands. Therefore, it provides a detailed representation of an imaged scene [12], where each pixel in an image contains a continuous spectrum of information across a range of wavelengths. HSI is used in various fields such as food quality and safety [13,14], and medical applications [15,16,17,18,19].

The medical field extensively researches HSI due to factors like multiple scattering and absorption from the light delivered to the tissue, stemming from the inhomogeneity of biological structures such as haemoglobin and melanin [20]. HSI can be utilised to diagnose different types of diseases that are typically in the outer layers of the human body, such as skin and breast cancer [21]. But considering the high costs of hyperspectral cameras, the technology is generally out of reach for commercial applications. Therefore, researchers have been exploring novel solutions for reconstructing hyperspectral images from RGB images.

An automatic facial skin defect detection system was proposed by [22]. This system first locates the facial region from an image. Then, the system extracts the region-of-interest (ROI) by using the empirical information of the facial features in the YCbCr and hue saturation value (HSV) colour spaces while removing the ears from the profile face. A support vector machine (SVM) classifier is used to classify potential defects into three categories, namely, normal, acne and spots. The researchers adopted a colour space based on YCbCr to detect potential skin defects. They found that acne has relatively high values in that colour space, which increases the accuracy of the SVM classifier.

Another research group proposed a system for the automatic detection and counting of acne in [23] where the input can be any part of the face with acne and lesions. The system first converts the input RGB image into a greyscale image. Next, the grey image is normalised and converted into an HSV image. The ROI is then obtained by subtracting the grey data from the HSV image, and with a specific threshold, the non-ROI features are eliminated and ROI spots are labelled and counted. Given that the properties of some facial problems may be similar to those of acne, the system has a high chance of false positives, which decreases its overall accuracy. Additionally, with this method, the colour, shape, and lighting conditions of acne have a great influence on the detection result.

Ref. [24] presented a Convolutional neural network (CNN)-based diagnostic approach for identifying facial acne that addresses the limitations of the earlier methods of [22,23] that failed to classify distinct forms of acne vulgaris precisely. The ROI was located with a skin detection method using a binary classifier and CNN that can extract features from input images, and then differentiate between skin and nonskin components through classification. Acne classification was achieved with a CNN model that extracts features from images and classifies them into seven categories. They then compared their feature extraction CNN model with a pre-trained VGG16 neural network and found that the pre-trained model performed better than their neural network because of their small dataset and the high similarity of their augmented data. Thus, they applied transfer learning for the pre-trained VGG16 with their dataset. This model performed feature extraction, and the features were regarded as inputs to the classifier model performing acne classification.

The researchers in [25] proposed a system for acne object detection with a model for grading acne severity. They used the Faster R-CNN architecture [26] with the ResNet50 backbone for the detection of four types of acne, namely, blackheads, whiteheads, papules, and pustules. The output of the model was then used as input for the severity grading model. The performance of the object detection model in detecting acne objects was assessed by using the average precision (AP) for each acne type and the mean average precision (mAP) for all four acne types. The object detection model achieved the highest AP for nodule/cyst lesions (0.68) and the lowest AP for blackhead/whitehead lesions (0.4). The mAP for all four acne types was 0.54, indicating overall moderate performance in detecting and counting acne lesions. The authors noted that additional labelled data can improve the mAP of the system and the precision of each type.

Ref. [27] examined two different models for acne detection, namely, Faster R-CNN and region-based fully convolutional network (R-FCN) [28]. The main goal of the study was to overcome the limitations of traditional acne detection methods, which are performed manually and are time-consuming. The dataset used in the study consisted of 871 annotated RGB images and was labelled by a dermatologist. The results indicated that the R-FCN model was more effective in detecting acne than Faster R-CNN. The R-FCN and Faster R-CNN models achieved mAPs of 28.3 and 23.3%, respectively. The authors stated that the proposed deep neural network-based method outperformed image processing methods based on other colour spaces.

Previous publications have proposed several technical solutions for the automatic detection of acne vulgaris. They have explored it either by using CNN or feature extraction, and most methods are based on colour spaces, such as RGB, YCbCr, and HSV. HSI captures a wider spectrum of light and can provide useful information that can be used in diagnosing different skin diseases in the outer layer of the human body. However, it is still not commercially used because of its high cost and the complexity of acquisition systems. Thus, no comprehensive system using hyperspectral images to automatically detect acne has been proposed.

The early detection of acne and other skin diseases is a crucial step to improve the quality of life of people suffering from acne [10,11]. The advantages of HSI in diagnosing different types of diseases but with high costs and complexity were the driving force behind exploring reconstructed HS images as a solution for medical diagnosis [29].

In our previous work in [29], a comparison between two different deep learning HSI reconstruction methods (HRNET and HSCNN-D) was carried out. The first step was data acquisition, where we captured HSI and RGB image pairs, the datasets then went through some pre-processing steps before being utilised to train HSI reconstruction models. The HSI images dataset consisted of hypercube data with a resolution of 512 × 512 pixels and 112 bands (512 × 512 × 112). As per our previous work, acne had a spectral signature deflected peak in a range of 600–650 nm, and since the utilised HSI camera (SPECIM FX10 [30]) records a high spectrum range (400–1000 nm) represented in 112 bands. Some of the spectrum bands shows unimportant information. Therefore, the hypercube spectral range was narrowed down from 112 bands to 25 bands (512 × 512 × 25) to cover the acne spectral signature range (570–690 nm). After that, a colour correction algorithm (Grey World Algorithm), was applied to the RGB dataset to maintain colour consistency across varying lighting conditions. Finally, data augmentation techniques were used on the datasets to artificially increase the dataset size. After pre-processing, several models with different hyperparameter configurations (batch size, colour correction, random cropping) were trained to identify the optimal reconstruction algorithm for acne hyperspectral images.

The study has shown that HRNET [31] architecture has outperformed HSCNN-D [32] architecture in the task of reconstructing hyperspectral images from RGB images. HRNET-5, which uses a small batch size, colour correction, and random cropping, has the lowest mean relative absolute error of 0.0433. Based on the final model (HRNET-5) that will be utilised in this study, and previous research [29,31], the model execution time was approximately 5.4 s running on a Nvidia GeForce RTX 2060 graphics card and AMD Ryzen 7 3700X Processor (4.2 GHz).

In this research, a novel method using the best-performing reconstructed HS images model obtained in [29] is proposed to detect acne vulgaris. The objective is to explore the feasibility of using reconstructed hyperspectral imaging for medical applications, in this case, specifically for acne detection.

To evaluate the performance of the suggested algorithm that uses the reconstructed HS images, several object detection networks will be trained on the three datasets (i.e., original HSI dataset, reconstructed HS dataset and RGB dataset) and different network backbones. This comparison demonstrates that the proposed acne detection algorithm, which utilises reconstructed HS images, could serve as a viable and cost-effective alternative compared to many other existing techniques that rely on RGB images. This paper will be organised as follows: Section 2 will discuss the system setup and the data preparation. Section 3 will outline the acne detection techniques that are based on RetinaNet, an object detection deep learning model. In Section 4, the best-performing acne detection algorithm based on a reconstructed HS dataset will be identified. Then a comparison of the performance of the proposed acne detection algorithm, which is based on the selected reconstructed HS images, with models that utilise RGB images and the original HS images, is presented. Finally, the conclusion and findings of the paper are summarised.

## 2. Materials and Methods

The main objective of the proposed work is to automate the process of the medical examination of acne, which would enable dermatologists to precisely evaluate the severity and localisation of the addressed issue. Additionally, the adoption of HSI and detection algorithms can improve diagnosis accuracy and overall workflow efficiency in busy healthcare centres.

The best-performing model identified by the previous study through a thorough analysis of the HS reconstruction algorithms models with different configurations [29] will be used in creating a third dataset (reconstructed HS images). Then by using the three different datasets (i.e., original and reconstructed HS datasets and RGB dataset), several detection models based on RetinaNet, an object detection algorithm, will be trained to detect acne regions, and the best-performing configuration will be identified.

### 2.1. System Setup and Data Preparation

**Figure 1:**
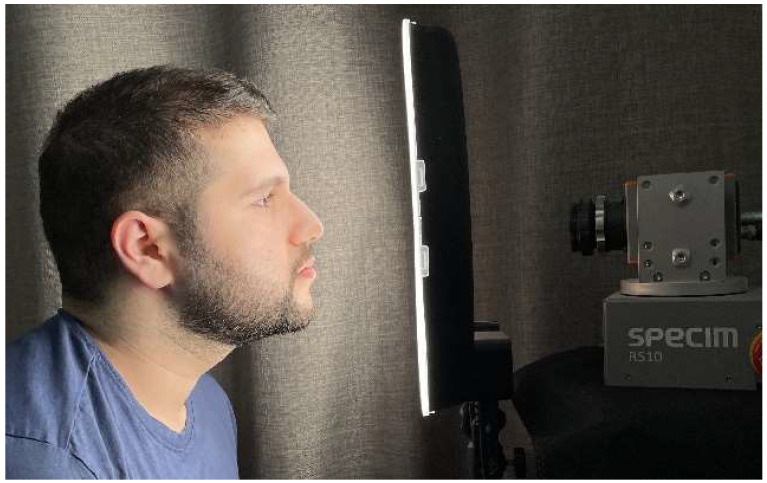
Data acquisition setup.

Data acquisition was performed in our previous study [29], and a SPECIM FX10 hyperspectral camera [30] was used to acquire the hyperspectral and RGB images. The camera has a spectrum range of 400–1000 nm represented in 112 bands. An LED ring light was used to evenly distribute light on a patient’s face, it was placed approximately 8–10 cm from the face as shown in Figure 1. The camera required approximately 15 s to capture the HS image because it is fixed on a rotary motor from the manufacturer

A total of 126 sample HS images from 38 patients were collected under the supervision of a dermatologist in accordance with Ajman University’s ethics committee standards under approval number 2021-IRG-ENIT-13.

Although a diverse dataset including various skin tones, angles, and positions of the face would typically be required for a more comprehensive deep learning model, in this study, a small dataset was collected and utilised. The participants were majorly light skin-toned, with a gender distribution of 45% female and 55% male.

The captured HS images acquired had a high spectrum range and some of the spectrum bands showed unimportant information. Therefore, in our previous study, the hypercube spectral range was narrowed down from 112 bands to 25 bands to cover only the acne spectral signature range.

**Figure 2:**
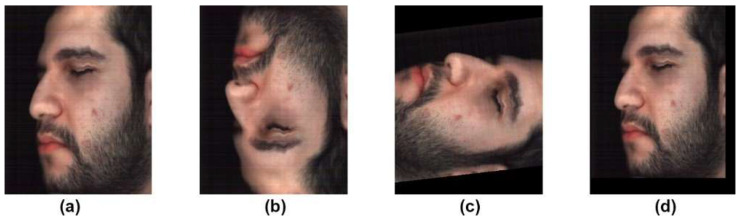
Examples of transformations. (**a**) Original image. (**b**) Flipping transformation. (**c**) Rotation transformation. (**d**) Padding transformation.

Additionally, data augmentation was performed on all three datasets to artificially increase the dataset size. As shown in Figure 2, only simple transformations were used in this study for the augmentation of the dataset (i.e., rotation, flipping, and padding).

### 2.2. Acne Region Detection

**Figure 3:**
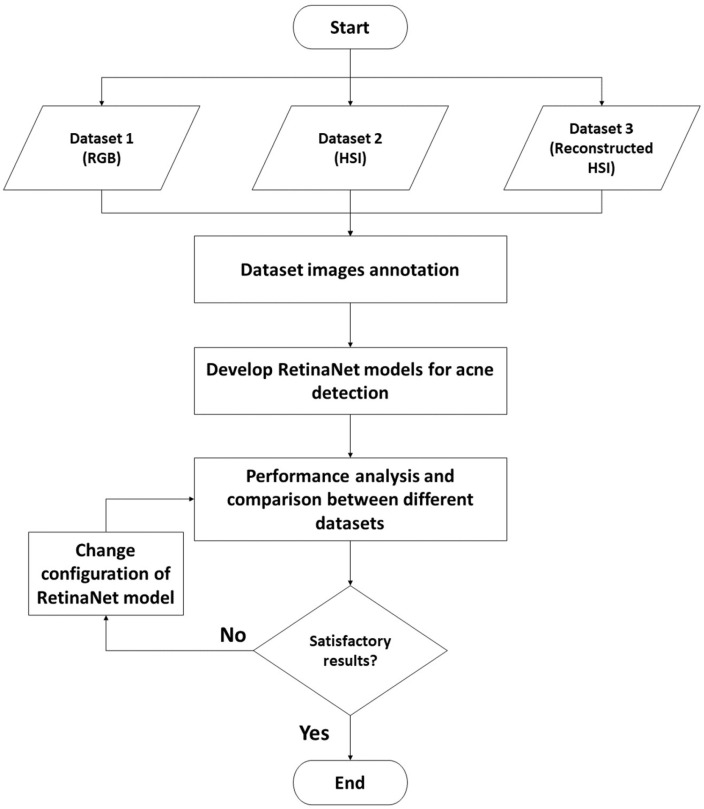
Flowchart for the process of acne detection models training.

The first step before training detection models was annotating images from all three datasets, as shown in Figure 3. This step will allow the model to accurately differentiate between acne spots and other skin spots that are not acne. The models were then trained using these datasets. By comparing the performance of object detection models trained on the original HS images, reconstructed HS images and RGB images, we can determine which type of input data is the most effective for acne detection. The importance of evaluating the performance of these models with different evaluation metrics will be covered. By comparing the performance of the models trained on the different datasets, the most effective approach for acne detection can be determined. This information will then be used in optimising the design of the acne detection system.

#### 2.2.1. Image Annotation

**Figure 4:**
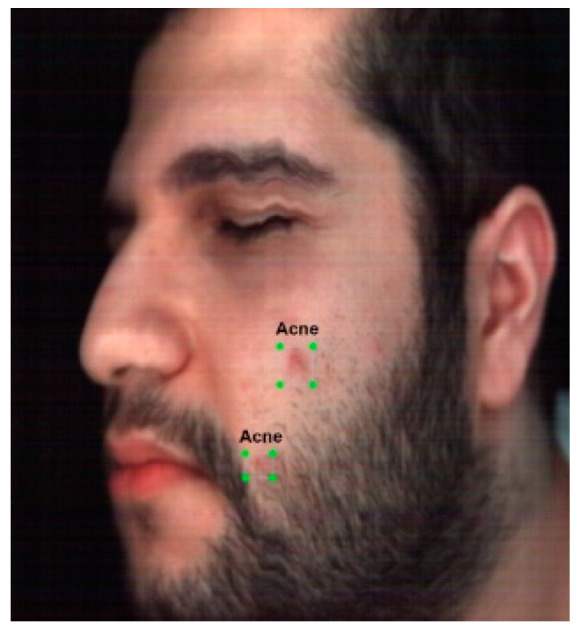
Annotated image from the dataset (Acne Annotations).

Object detection is a type of supervised learning. Thus, a dataset should be labelled with the position of the objects of interest (acne regions in every image). Labelling is typically carried out by manually annotating the dataset by labelling the drawn bounding boxes around every instance of an object (acne). There are different methods for labelling datasets, and one common method is a graphical annotation tool such as Label Studio [33]. This open-source graphical data labelling tool was utilised to label the three datasets. The Information of each image was then saved in an XML file. After labelling was completed, the datasets were augmented using flipping, rotation and padding, and the dataset size was increased to approximately 500 images as shown in Figure 4, an example of an annotated image. This process was assessed and monitored by a dermatologist, and the annotated regions that were valid acne regions were used for acne detection.

#### 2.2.2. Acne Detection Models

Acne detection is a specialised task within the field of object detection and image segmentation. There are various algorithms for object detection; one-stage algorithms, such as YOLO [34], perform detection in a single pass and are known for their speed and efficiency. While multi-stage algorithms like Faster R-CNN [26], involve a more complex process with separate stages for generating region proposals and refining detections. In this study, we have used RetinaNet [35], which balances speed and accuracy by utilising a feature pyramid network and focal loss to address class imbalance, making it particularly effective for detecting small and dense objects like acne.

RetinaNet [35] is a one-stage algorithm for object detection designed to be more efficient and accurate than two-stage object detection algorithms. This is achieved by using a single network to simultaneously predict object class probabilities and the bounding box coordinates, unlike in two-stage algorithms where two separate networks are used.

**Figure 5:**
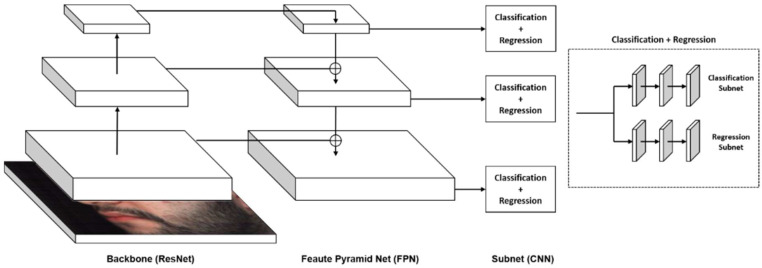
Illustration of the RetinaNet network structure.

The structure of RetinaNet is shown in Figure 5. RetinaNet is composed of a backbone and feature pyramid net (FPN) structures.

The ResNet backbone is used to calculate the convolutional feature maps at different scales, which are used as inputs to the rest of the network to predict object classes and bounding boxes. The feature maps are computed using a series of convolutional layers and residual connections, which allow the network to capture increasingly complex features as it processes the input image.

The bottom-up pathway (ResNet Backbone) in a RetinaNet model performs the process of computing the feature maps at different scales, starting from the lowest resolution and working up to the highest resolution. This allows the network to capture information at different levels of detail, which is important for accurately identifying objects in an image. The top-down pathway (FPN) performs the process of upsampling feature maps from higher pyramid levels and combines it with feature maps from lower pyramid levels to produce a final set of feature maps that can be used to make object detection predictions.

A CNN network that processes the feature maps and produces predictions is connected to each level of the pyramid. The CNN network typically comprises two independent subnetworks, one for the classification used in predicting the class label of an object in an image, and the other for regression, which is used to predict the location of the object in the image.

One of the main advantages of RetinaNet is that it utilises focal loss (FL) [35] function that addresses class imbalance during training. In object detection tasks, an extremely large number of negative examples, regions that do not contain any objects, was common. This can lead to a model that more accurately predicts negative examples and struggles to accurately predict positive examples, i.e., regions that do contain objects (acne). What FL does is down-weight the loss for negative examples while up-weighting it for positive examples, enabling the object detection model to focus on positive examples and improve the ability to predict them. As a result, RetinaNet can achieve state-of-the-art performance on a variety of object detection benchmarks.

Given that the RetinaNet algorithm is designed to take RGB images (3 bands) as input, and the HS images (25 bands) have a higher dimension than RGB, the original RetinaNet model must be modified. The input layer should be adjusted and additional convolutional layers are required to be added to handle the additional bands in a HS image. The top layers of the pre-trained RetinaNet backbone are shown in Table 1.

For the pre-trained model to be able to accept the HS images, the input layer was modified to accept 25 bands instead of three, as shown in Table 2. Two convolutional layers with filter sizes 32 and 64 were also added after the input. These convolutional layers allowed the model to extract increasingly complex features from the HS data. Additionally, a batch normalisation layer was added, which can help to stabilise the training process and improve the generalisation of the model. That, in turn, can reduce the risk of overfitting and improve the model’s performance on unseen data.

The models were built based on PyTorch framework (Python), and the training was performed on a Nvidia GeForce RTX 2060 graphics card and AMD Ryzen 7 3700X Processor (4.2 GHz). Nine models were trained with different ResNet backbones, and different datasets, i.e., original HS dataset, reconstructed HS dataset, and RGB dataset as tabulated in Table 3.

All nine configurations use the same hyperparameters (Table 4) of batch size, learning rate, and optimiser (Adam). The models were pre-trained on the MS COCO dataset [36], which consists of RGB images. After that, using transfer learning, the models were fine-tuned to the acne datasets. The datasets were uniformly divided based on the samples from patients in a ratio of 60% for the training set, 20% for the validation set and 20% for the testing set.

#### 2.2.3. Acne Model Evaluation

To evaluate the performance of the acne detection models, the standard evaluation metrics for object detection models were used. Based on the output of the model, four outcomes are possible: true positive (TP) if an acne region is detected correctly, false positive (FP) if the acne is detected but no acne is present within the region, false negative (FN) if acne region is detected but the model predicts acne that is not present, and true negative (TN) if acne is not present. These metrics were used in calculating the precision, recall, F1 score, and mean average precision (mAP).

Precision is a metric for the number of correct positive predictions, and it is calculated as the ratio of correctly predicted targets divided by the number of all positive predicted targets, as shown in (1). Recall is the number of correct positive predictions out of all the positive predictions that have been made, as shown in (2).

(1) $$Precision=\frac{TP}{TP+FP}$$

(2) $$Recall=\frac{TP}{TP+FN}$$

The F1 score is the harmonic average of the precision and recall metrics. It is used in comparing the performance of detection models and can be calculated using (3)

(3) $$F_{1}=\frac{2\times Precision\times Recall}{Precision+Recall}$$

The mAP is used in evaluating the overall performance of object detection models. It is equal to the mean value of the summation of all average precision values, which is defined as the area under the precision–recall curve and calculated using (4) and (5).

(4) $$AP=\int_{0}^{1}P\left(r\right)dr$$

(5) $$mAP=\frac{1}{n}\sum_{i=1}^{n}AP_{i}$$

#### 2.2.4. Comparison with Existing Methods

The performance of the proposed HSI acne detection system will be compared to existing methods that utilise RGB images. The two methods introduced [25,27] are developed and trained by using the RGB dataset and then evaluated by using the same metrics as those described in the Section 2.2.3 for comparison with the proposed system. These two methods are chosen because they have higher performance and the ability to detect multiple acne regions and their positions compared with the other methods discussed in the Section 1.

The Faster R-CNN model is built based on the PyTorch framework based on [25]. Training is performed on an Nvidia GeForce GTX 2060 graphics card and AMD Ryzen 7 3700X Processor @ 4.2 GHz. The model uses the same hyperparameters shown in Table 4 and is pre-trained on the MS COCO dataset [36]. Then the models were fine-tuned to the acne datasets. The datasets used are the same divided datasets from Section 3.2. And the R-FCN model based on [27] is built based on the TensorFlow framework. Training is performed with an Nvidia GeForce GTX 2060 graphics card and AMD Ryzen 7 3700X Processor @ 4.2 GHz. The backbone used for the model was ResNet-101, with batch size 2 and a learning rate of $1\times 10^{-5}$.

## 3. Results

This section will present the results and analysis of the proposed acne detection system. After using the best-performing RGB to HSI model to create a third dataset consisting of reconstructed HS images, several acne detection models were trained, with all three datasets (original HS, reconstructed HS and RGB), and different network backbone depths (ResNet18, ResNet34, and ResNet50). The results of these acne region detections are presented in Section 3.1. Additionally, the results of the performance comparison between the proposed method and two existing methods for acne detection are presented in Section 3.2.

### 3.1. Results of Acne Region Detection

As previously discussed, because of the difficulty in acquiring the HS images of acne and the lack of available datasets, 126 images (283 acne regions) were collected under the supervision of a dermatologist and then artificially augmented to approximately 500 images (1100 acne regions) that were divided into three different datasets (60% for training, 20% for validation and 20% for testing).

The four metrics previously discussed (precision, recall, F1 score and mAP) were used in evaluating the results of the acne region detection models. The final model’s evaluation for all configurations is tabulated in Table 5.

**Figure 6:**
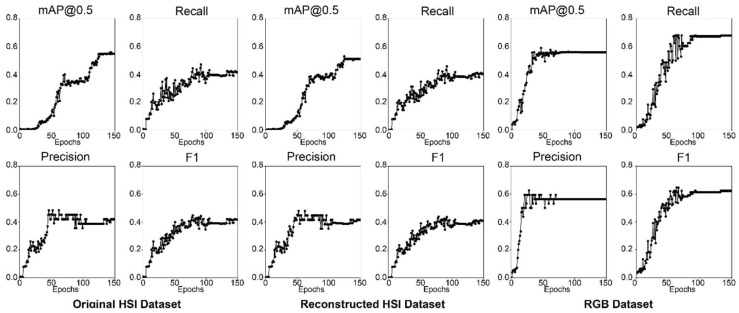
Graphs of the ResNet18 acne detection models performance metrics.

**Figure 7:**
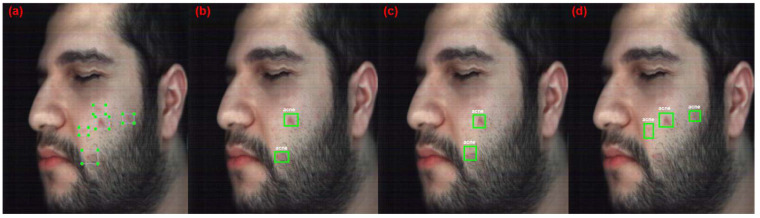
Detection results of the Acne models with ResNet18. (**a**) Original annotated image (**b**) Original HSI dataset model. (**c**) Reconstructed HSI dataset model. (**d**) RGB dataset model.

Figure 6 shows the four metrics over all epochs during training for ResNet18 models with different datasets (original HS, reconstructed HS, and RGB). The results show that the RGB model stops improving at epoch 70, and the original and reconstructed HS models require more training epochs to reach better results. Additionally, the results show that the model with the RGB dataset has the highest overall performance, with an F1 score of 62.35%, whereas the HS models (original and reconstructed) have a lower performance, with F1 scores of 41.73% and 38.12%, respectively. The HS datasets performed poorly compared with the RGB dataset probably due to the relatively shallow network architecture of the ResNet18 backbone, which has 18 layers only. This number of layers may be insufficient to capture the full range of features in the HS dataset, which can be considered highly complex. Figure 7 shows the detection results with a detection score of more than 50% for the best-performing models from ResNet18. Both HS models have low performance since they were able to only detect two acne regions (40%) in the test image, which has five acne regions annotated, whereas the RGB model detects three acne regions (60%).

The ResNet18 model performance is relatively close across the three datasets, i.e., (RGB, Original HS, and Reconstructed HS), but the RGB dataset has the highest metrics (precision, recall, and mAP) values. While the original HS performs better than the reconstructed HS dataset but both have lower performance than the RGB dataset.

**Figure 8:**
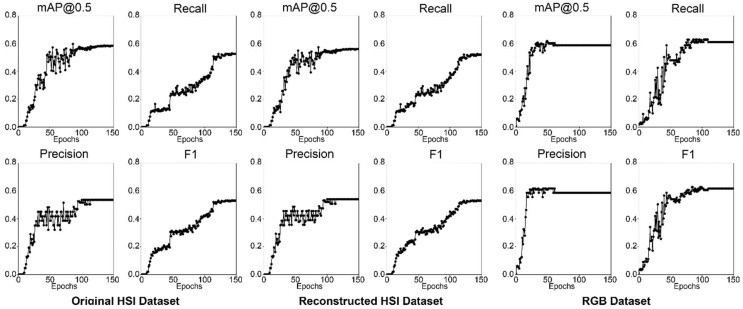
Graphs of the ResNet34 acne detection models performance metrics.

**Figure 9:**
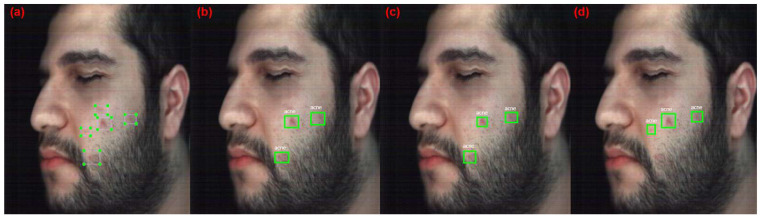
Detection results of the Acne models with ResNet34. (**a**) Original annotated image (**b**) Original HSI dataset model. (**c**) Reconstructed HSI dataset model. (**d**) RGB dataset model.

The four metrics results over all epochs during training for the ResNet34 models are shown in Figure 8. Similar to the ResNet18 models, the RGB model stops improving at epoch 70, whereas both HS models kept improving until epoch 150. The results show that the RGB model has the highest overall performance, with an F1 score of 61.83% as tabulated in Table 5. However, the HS models’ performance improved compared to the ResNet18 backbone, with F1 scores of 53.39% and 53.38% for the original and reconstructed HS models, respectively. This improvement in performance is probably due to ResNet34 being a deeper architecture than ResNet18 and having 34 layers, which may be better suited for the complex features present in HS datasets. Figure 9 showcases the ResNet34 acne detection model results with a score of more than 50% on a test image. All three models have better performance than ResNet18, with the HS models detecting three acne regions. The RGB model also detects three acne regions. The results for the original HS and reconstructed HS are relatively similar, and the difference in performance is minor, suggesting that the reconstructed HS does not significantly impact the performance of the acne detection model. In general, models with the ResNet34 backbone have better performance than ResNet18 and have high precision, recall, and mAP values. The RGB dataset has the highest performance in all metrics, followed by the reconstructed HS dataset.

**Figure 10:**
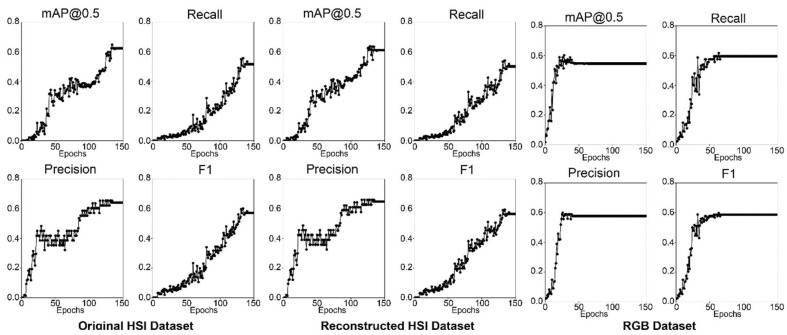
Graphs of the ResNet50 acne detection models performance metrics.

Figure 10 showcases the four metrics results over all epochs during training for the ResNet50 models. Similar to ResNet18 and ResNet34, the RGB model stops improving after epoch 70 while both HS datasets require additional training epochs to produce better results. This finding indicates that the HS dataset models require more training to reach optimal performance. The probable reason is that the HS input data is larger than the RGB data, and a large input size requires a model to learn complex patterns and features. As shown in Figure 6, Figure 8 and Figure 10, the performance of the RGB dataset models increases more linearly than the HS datasets, with the HS datasets (original and reconstructed) showing exponential increase.

The results show that the performance of the ResNet50 models was greatly improved in comparison with the ResNet18 and ResNet34 models, particularly the HS models. The original and reconstructed HS models have F1 scores of 60.27% and 59.55%, whereas the RGB model has an F1 score of 58.64% as tabulated in Table 5. The RGB model has lower performance than the HS models, which is different from the results obtained with ResNet34 and ResNet18. Thus, improvements in the performance of the HS datasets can be attributed to the deeper architecture of the ResNet50, which has 50 layers of neural network architecture, which can capture complex features in the HS dataset and thereby improve performance for acne detection.

**Figure 11:**
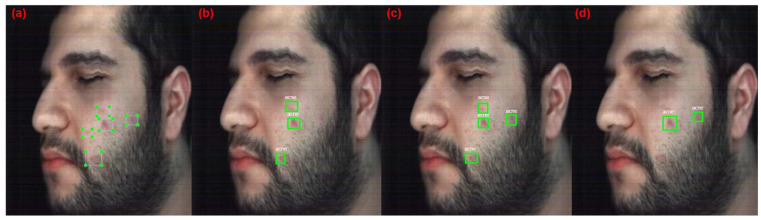
Detection results of the Acne models with ResNet50. (**a**) Original annotated image (**b**) Original HSI dataset model. (**c**) Reconstructed HSI dataset model. (**d**) RGB dataset model.

Figure 11 showcases the results with a detection score of more than 50% for the ResNet50 acne detection models on a test image, and the HS models have better performance than previous backbone networks (ResNet18 and ResNet34). The original HS model detects three acne regions, whereas the reconstructed HS model detects four acne regions. However, the RGB model detects only two acne regions, which is lower than the ResNet34 results. These results show that the original and reconstructed HS Images were able to detect one acne region which was never detected by the RGB model, because it is not apparent on the skin, but diagnosed by the dermatologist as an underdeveloped acne region. Thus, the use of HSI can aid in the detection of underdeveloped acne that has yet to be apparent on the skin.

The ResNet50 backbone and original HS dataset have the highest recorded mAP values (64.81% and precision of 65.48%; Table 5), suggesting that the highest average precision in detecting acne can be obtained by using HS images. Additionally, the second highest mAP is produced by the reconstructed HS dataset with the ResNet50 backbone model (63.52%), and it has a higher precision (66.14%) than the original HS. This result confirms that reconstructed HS can be as accurate as the original HS dataset for acne detection.

Additionally, the results of the acne detection models indicate that the performance of the models is impacted by the backbone and dataset. The models with higher ResNet backbones performed better with HS datasets but had the opposite effect on the RGB dataset models. The RGB dataset and ResNet34 backbone have the best performance among the RGB dataset models. The RGB dataset with the ResNet18 backbone has extremely high recall and precision of 67.84% and 56.32%, respectively, compared with the original and reconstructed HS datasets models with the same backbone (ResNet18). Recall can be regarded as a measure of quantity, and a high recall implies that the model is returning the most results regardless of whether they are correctly identified or not. The RGB dataset performs well with a low ResNet backbone in terms of precision and recall but has a lower mAP and is outperformed by the HS datasets in high backbone depths (ResNet34 and ResNet50). The reconstructed HS and ResNet50 model achieved the highest precision of 66.14%, indicating that this model makes fewer false positive detections for acne and can identify real acne regions accurately.

The dataset used, consisting of approximately (1100 acne regions), can be considered a decent size for a medical image dataset, given the difficulty in acquiring HS images. However, it is still smaller compared to open-source large medical datasets, which can contain tens of thousands of images. Therefore, collecting and annotating a larger dataset of HS acne images with different types of acne can lead to accurate and robust models that can diagnose different types and severity of acne. Considering that HS can be very challenging to use in practice because it requires specialised hardware and is extremely expensive, reconstructed HS can be a viable alternative because it reconstructs images by using algorithms and typical RGB images. While the resulting reconstructed images may not be an exact copy of the true HS, the results suggest that reconstructed HS is an accurate representation and can provide useful information just like the original HS image. This can serve as a catalyst for using hyperspectral imaging more in the medical field. Since the use of hyperspectral imaging in medical applications has been limited due to the high cost and complexity of the equipment required, utilising reconstructed HS is beneficial as it provides a cost-effective and accessible way of acquiring HS data without compromising the accuracy of the acne detection model.

This method has high potential in the diagnosis of a wide range of medical conditions, where there is no longer a need to have specialised HSI hardware after the initial development stage. Using low-cost off-the-shelf digital cameras and HSI reconstruction algorithms will allow the easy integration of HSI into the clinical workflow. But the development of a user-friendly interface for the clinical staff and the willingness of the clinicians to adopt such technology will also be a requirement. While the proposed method has the potential of being a tool for dermatologists to improve the accuracy of acne diagnostics and reduce the time and extensive effort in manually detecting acne, the proposed method will require to be approved by the respective regulatory agencies before it can be used in clinical settings.

Additionally, since the reconstructed HS images are a viable alternative to the original HS images, this can allow the creation of new datasets of HS images with only RGB images and the HSI reconstruction models, without the requirement of capturing HS images using HSI cameras. This can assist in creating new HSI databases for use in the medical field, where capturing HSI images may not be feasible.

### 3.2. Performance Comparison

Two existing methods for acne detection [25,27] were developed, trained with the same RGB datasets, and compared with the proposed system to provide a comprehensive and fair evaluation of the proposed system’s performance. As discussed in the introduction, Huynh et al. (2022) utilised Faster R-CNN with the ResNet50 backbone and RGB images for acne detection, whereas Rashataprucksa et al. (2020) developed an RGB acne detection system with the F-RCN architecture and ResNet101 backbone.

The four metrics previously discussed in Section 2.2.3, namely, precision, recall, F1 score, and mAP, are used to evaluate the results of models for acne region detection. The final developed models of Huynh et al., (2022) [25], i.e., (Faster R-CNN with ResNet50 and RGB), Rashataprucksa et al., (2020) [27] (i.e., F-RCN with ResNet101 and RGB), and the proposed system utilising RetinaNet with reconstructed HSI were evaluated to assess the performance of the models for acne region detection. The results are tabulated in Table 6.

The results illustrate that the proposed system utilising RetinaNet with reconstructed HS demonstrates its superiority by achieving higher performance than the existing methods based on Faster R-CNN (RGB) and F-RCN (RGB). These results validate the efficacy of utilising reconstructed HSI for acne detection, highlighting the potential of HSI in improving the accuracy and reliability of acne detection systems.

The results show that the proposed method, which utilises RetinaNet with reconstructed HSI, outperforms the existing methods (Faster R-CNN and F-RCN) in terms of precision. The proposed method achieves a precision of 66.14%, which surpasses the precision of Faster R-CNN (52.44%) and F-RCN (56.61%). This finding highlights the superior accuracy of the proposed model in correctly identifying acne regions. Table 6 illustrates that amongst the three models, the proposed method achieves the highest mAP of 63.52%. By contrast, F-RCN has an mAP of 59.43% and Faster R-CNN has the lowest mAP of 56.28%.

Additionally, the performances of the Faster R-CNN and F-RCN models that use RGB images are close to the results obtained by the RetinaNet model that uses ResNet34 and RGB images with a precision of 58.83% (Section 3.1). These findings indicate that the choice of algorithm and backbone architecture plays an important role in the models’ performance. Although the Faster R-CNN and F-RCN methods perform well in acne detection, they are still outperformed by the proposed HS method. This result suggests that the proposed method, which utilises reconstructed HS, possesses the ability to identify acne regions with higher accuracy and precision. The results highlight the potential advantages of utilising HSI data in acne detection. The proposed method achieves higher performance than the existing RGB-based models by leveraging the spectral information captured by HSI. The ability of the proposed method to identify acne regions accurately indicates its potential for improving the accuracy and reliability of acne detection systems.

But the maximum precision attained might still be considered low compared to what would be required for an efficient diagnosis of acne. Hence, to improve accuracy, more diverse datasets are required, and it may be beneficial to explore different advanced deep-learning techniques.

## 4. Discussion

The main objective of this research is to develop a deep learning-based acne region detection method that utilises reconstructed HSI. The best-performing model for reconstructing acne hyperspectral images from the previous study (i.e., HRNET) is utilised to create a dataset consisting of reconstructed HS images, which is then used for the acne detection system. This dataset serves as the foundation for the development of an acne detection algorithm based on the RetinaNet architecture, which is set as a second research objective. By leveraging the reconstructed HS images, the proposed method aims to improve the accuracy and effectiveness of acne detection compared to existing methods.

Several configurations of RetinaNet models with different network backbones (i.e., ResNet18, ResNet34, and ResNet50) and datasets (i.e., RGB, original HSI, and reconstructed HSI) are analysed. The results demonstrate that although the RGB dataset has good performance, it is still outperformed by the HSI datasets with high backbone depths (i.e., ResNet50). Amongst all configurations, the ResNet50 model with the original HSI dataset exhibits the highest performance with an mAP of 64.81%. Meanwhile, the ResNet50 and reconstructed HSI model has the second-highest performance with an mAP of 63.52%. This finding demonstrates that reconstructed HS images can provide higher performance than RGB images and present useful spectral information similar to the original HS images.

This study has investigated and compared the performance of the proposed method (i.e., RetinaNet with ResNet50 and reconstructed HSI) and two existing methods (i.e., Faster R-CNN with ResNet50 and F-RCN with ResNet101) to prove the capability of the proposed method. The evaluation performed in this work is based on measurement metrics, such as precision, recall, F1 score, and mAP. The results show that the proposed method outperforms the other two existing methods for acne detection. The proposed method of RetinaNet with ResNet50 and reconstructed HSI produces an mAP of 63.52%, which is higher than the mAPs of 56.28% and 59.43% produced by Faster R-CNN and F-RCN, respectively. These findings indicate that this study has successfully developed a reliable acne detection system using reconstructed HS images.

While the results for the proposed reconstructed HSI acne detection model are promising, one of the main limitations is the small sample size, where the trained model may not have the same generalisability to a more diverse population of patients. Therefore, a large diverse dataset of HS images can improve the generalisability of the model. Additionally, the proposed method currently only detects acne without identifying the type or severity of the detected acne, this can be achieved by creating an HSI database with different types of acne labelled by a dermatologist.

Additionally, the HSI models required more time until convergence, unlike the RGB models. This might be because of the complexity of the spectral information in HSI, which requires more time for the model being trained to reach convergence. But the potential for achieving higher precision in medical imaging and analysis with HSI can be a reason to justify the additional resources and training duration required. Future research efforts should focus on optimising training strategies and further validating the efficacy of HSI in enhancing diagnostic capabilities across various medical conditions

The findings and insights gained from this study can pave the way for future research in the field of medical research using reconstructed HSIs. Some recommendations for future research with a focus on areas that can benefit from the utilisation of reconstructed HSI.

1. Investigate the development of algorithms that use reconstructed HSI for different types of acne lesions, such as comedones, papules, and pustules. Such an investigation can involve the analysis of HSI data to identify unique spectral signatures associated with each acne type.
2. Apply reconstructed HSI techniques to detect and classify various medical conditions beyond acne. Developing specialised algorithms based on reconstructed HSI can enhance diagnostic accuracy and improve treatment monitoring without the requirement for specialised HSI equipment.

## 5. Conclusions

In this research, a new efficient acne detection technique based on reconstructed HS images and the RetinaNet network was presented. The best-performing model from the previous study was used in creating a dataset of reconstructed HS images for the acne detection model. To evaluate the performance of the proposed acne detection algorithm, several detection models based on the RetinaNet object detection algorithm were presented and compared. Three different datasets namely the original HS, reconstructed HS, and RGB with different backbone networks (i.e., ResNet18, ResNet34, and ResNet50) were used for the acne detection models. The results suggest that by using RetinaNet with ResNet50 backbone, the HS images (original and reconstructed) that capture a wider range of spectral information than RGB images produce higher performance for acne detection. The proposed technique of RetinaNet with ResNet50 and reconstructed HS images produces an mAP of 63.52% compared to the other two RGB acne detection techniques analysed, with an mAP of 56.28% and 59.43%. The findings in this study could potentially catalyse the use of HSI in the medical field. By utilising reconstructed HS images, the benefits of HS data can be acquired in a cost-effective and accessible manner, without sacrificing the accuracy of the acne detection model. This research has significant implications for the medical field, as it enables the use of HSI in the diagnosis of acne and potentially other medical conditions. While RGB images are easy to capture, relying solely on RGB may limit the ability to diagnose acne that is not visible on the skin surface. The proposed method has a high potential for accurately diagnosing acne and potentially a wide range of medical conditions.

## Figures and Tables

**Table 1:** Pre-trained RetinaNet Backbone Top layers.

Layer Name	Type of Layer
Input (512 × 512) × 3 (RGB Image)	
bn1	BatchNorm2d
Relu	ReLU
layer1 Sequential—BasicBlock-0	
conv1	Conv2d-64
bn1	BatchNorm2d
Relu	ReLU
conv2	Conv2d-64
bn2	BatchNorm2d

**Table 2:** Modified RetinaNet Backbone Top layers for HSI.

Layer Name	Type of Layer
Input (512 × 512) × 25 (Hyperspectral Image)	
bn1	BatchNorm2d
Conv2	Conv2d-32
bn2	BatchNorm2d
Conv3	Conv2d-64
bn3	BatchNorm2d
relu	ReLU
layer1 Sequential—BasicBlock-0	
conv1	Conv2d-64
bn1	BatchNorm2d
relu	ReLU
conv2	Conv2d-64
bn2	BatchNorm2d

**Table 3:** Acne detection model configurations.

No.	Dataset	Algorithm	Backbone Network (Depth)
1	Original HSI	RetinaNet	ResNet18
2	Reconstructed HSI	RetinaNet	ResNet18
3	RGB Images	RetinaNet	ResNet18
4	Original HSI	RetinaNet	ResNet32
5	Reconstructed HSI	RetinaNet	ResNet32
6	RGB Images	RetinaNet	ResNet32
7	Original HSI	RetinaNet	ResNet50
8	Reconstructed HSI	RetinaNet	ResNet50
9	RGB Images	RetinaNet	ResNet50

**Table 4:** Hyperparameters configuration.

Hyperparameter	Value
Batch size	16
Optimiser	Adam
Learning rate	1 × 10^−5^

**Table 5:** Evaluation results of the acne detection models.

No	Dataset	Backbone Network	Precision (%)	Recall (%)	mAP (%)	F1 (%)
1	Original HSI	ResNet18	41.94	41.53	55.69	41.73
2	Reconstructed HSI	ResNet18	38.71	37.55	52.87	38.12
3	RGB Images	ResNet18	56.32	67.84	55.75	62.35
4	Original HSI	ResNet34	53.71	53.08	58.02	53.39
5	Reconstructed HSI	ResNet34	54.25	52.55	57.38	53.38
6	RGB Images	ResNet34	58.83	61.14	58.83	61.83
7	Original HSI	ResNet50	65.48	55.83	64.81	60.27
8	Reconstructed HSI	ResNet50	66.14	54.16	63.52	59.55
9	RGB Images	ResNet50	57.74	59.58	54.63	58.64

**Table 6:** Evaluation results of the proposed and existing acne detection models.

No.	Authors	Dataset	Algorithm	Precision (%)	Recall (%)	mAP (%)	F1 (%)
1	Proposed system	Reconstructed HSI	Retinanet with ResNet50	66.14	54.16	63.52	59.55
2	Huynh et al., (2022) [25]	RGB	Faster R-CNN with ResNet50	52.44 *	56.42 *	56.28 *	54.35 *
3	Rashataprucksa et al., (2020) [27]	RGB	F-RCN with ResNet101	56.61 *	61.68 *	59.43 *	59.03 *

* Results of the developed model based on the author’s proposed method.

## Data Availability

The datasets presented in this article are not readily available because they contain sensitive personal information. Requests to access the datasets should be directed to Ali Mohammed Ridha.
